# Supplementary material for: Combined Prospective Seroconversion and PCR Data of Selected Cohorts Indicate a High Rate of Subclinical SARS-CoV-2 Infections—an Open Observational Study in Lower Saxony, Germany
Source: Microbiol Spectr. 2022 Feb 16;10(1):e01512-21. doi: 10.1128/spectrum.01512-21 (PMC8849099; doi:10.1128/spectrum.01512-21)
Supplement: SUPPLEMENTAL FILE 1 — Supplemental material. Download SPECTRUM01512-21_Supp_1_seq10.pdf, PDF file, 0.1 MB [file spectrum01512-21_supp_1_seq10.pdf]

Supplement

Tables

Table S 1: Extract of the questionnaires subjects were inquired to complete before a throat swab (I), before a blood draw (II) or additionally before a blood drawn after January 2021 (III). The questionnaires refer to the beginning of the corona crisis and ask whether symptoms or risk regarding SARS-CoV-2 infection were ever present.

	**#**	**Question**	**Answer options**
O	1	Birthdate	date (dd.mm.yyyy)
	2	Gender	Male/female/diverse
	3	Are you working in one of the following areas/facilities?	Infotext
	4	Medical area	Free text field
	5	Community facility (school, kindergarten, nursing home, university)	Free text field
	6	Have you had contact to a confirmed SARS-CoV-2 case?	Yes/no/unknown
	7	Have you had one or more of the following symptoms within the last 14 days? If yes, state in each case for how many days.	Infotext
	8	Fever	Number
	9	Shivering	Number
	10	Increased fatigue or a significantly lower ability to work under pressure	Number
	11	Myalgia	Number
	12	Headache	Number
	13	Sore throat	Number
	14	Loss of taste and smell	Number
	15	Have you ever been diagnosed with any of the following chronic disease by a physician?	Infotext
	16	Chronic lung disease	Yes/no/unknown
	17	Diabetes	Yes/no/unknown
	18	Heart disease	Yes/no/unknown
	19	Adiposity	Yes/no/unknown
	20	Chronic disease of bowels	Yes/no/unknown
	21	Do you smoke?	Yes/no
	22	Are you currently taking cortisone (in tablets)?	Yes/no/unknown
	23	Are you currently taking immunosuppressants?	Yes/no/unknown
OO#	23	Have you experienced symptoms that you might now interpret as typical of a SARS-CoV-2 infection since the presumed onset of the Corona crisis in Germany (since approximately December 2019 or January 2020)?	Yes/no/unknown

	24	Which symptoms exactly?	Infotext
	25	Fever and feeling sick	Yes/no
	26	cough, cold	Yes/no
	27	Severe fatigue and decrease in performance	Yes/no
	28	Diarrhoea	Yes/no
	29	Sore throat	Yes/no
	30	Headache	Yes/no
	31	Pain in the limbs	Yes/no
	32	Test or smell disorder	Yes/no
	33	Shortness of breath	Yes/no
OOO#	34	Have you ever been tested positive for SARS-CoV-2 infection in the past?	Yes/no
	35	If yes: when was the test performed?	date (dd.mm.yyyy)
	36	Have you had the following symptoms as part of your infection? (0=not at all, 5=very strong)	Infotext
	37	Fever and feeling sick	number
	38	Cough, sore throat	Number
	39	Cold	Number
	40	Severe fatigue and decrease in performance	Number
	41	Diarrhoea	Number
	42	Headache	Number
	43	Pain in the limps or joints	Number
	44	Taste or smell disorders	Number
	45	Breathlessness and shortness of breath	Number
	46	Skin rash	Number
	47	Lung inflammation	Number
	48	In the meantime, have you been vaccinated against SARS-CoV-2?	Yes/no
	49	If yes: when have you been vaccinated (first time)?	date (dd.mm.yyyy)
	50	If yes: have you been vaccinated with a viral vaccine or mRNA agent?	mRNA/Vector/Unknown
	51	If yes: please tell us the name of vaccine (e.g., Biontech, Moderna, Pfizer)	Free text field

#

#
